# CRISPR-iPAS: a novel dCAS13-based method for alternative polyadenylation interference

**DOI:** 10.1093/nar/gkac108

**Published:** 2022-02-22

**Authors:** Shuye Tian, Bin Zhang, Yuhao He, Zhiyuan Sun, Jun Li, Yisheng Li, Hongyang Yi, Yan Zhao, Xudong Zou, Yunfei Li, Huanhuan Cui, Liang Fang, Xin Gao, Yuhui Hu, Wei Chen

**Affiliations:** Shenzhen Key Laboratory of Gene Regulation and Systems Biology, Department of Biology, School of Life Sciences, Southern University of Science and Technology, Shenzhen 518055, China; Computational Bioscience Research Center (CBRC), Computer, Electrical and Mathematical Sciences and Engineering Division, King Abdullah University of Science and Technology (KAUST), Thuwal, Saudi Arabia; Shenzhen Key Laboratory of Gene Regulation and Systems Biology, Department of Biology, School of Life Sciences, Southern University of Science and Technology, Shenzhen 518055, China; Shenzhen Key Laboratory of Gene Regulation and Systems Biology, Department of Biology, School of Life Sciences, Southern University of Science and Technology, Shenzhen 518055, China; Shenzhen Key Laboratory of Gene Regulation and Systems Biology, Department of Biology, School of Life Sciences, Southern University of Science and Technology, Shenzhen 518055, China; Academy for Advanced Interdisciplinary Studies, Southern University of Science and Technology, Shenzhen 518055, China; Shenzhen Key Laboratory of Gene Regulation and Systems Biology, Department of Biology, School of Life Sciences, Southern University of Science and Technology, Shenzhen 518055, China; Shenzhen Key Laboratory of Gene Regulation and Systems Biology, Department of Biology, School of Life Sciences, Southern University of Science and Technology, Shenzhen 518055, China; Shenzhen Key Laboratory of Gene Regulation and Systems Biology, Department of Biology, School of Life Sciences, Southern University of Science and Technology, Shenzhen 518055, China; Shenzhen Key Laboratory of Gene Regulation and Systems Biology, Department of Biology, School of Life Sciences, Southern University of Science and Technology, Shenzhen 518055, China; Shenzhen Key Laboratory of Gene Regulation and Systems Biology, Department of Biology, School of Life Sciences, Southern University of Science and Technology, Shenzhen 518055, China; Shenzhen Key Laboratory of Gene Regulation and Systems Biology, Department of Biology, School of Life Sciences, Southern University of Science and Technology, Shenzhen 518055, China; Academy for Advanced Interdisciplinary Studies, Southern University of Science and Technology, Shenzhen 518055, China; Shenzhen Key Laboratory of Gene Regulation and Systems Biology, Department of Biology, School of Life Sciences, Southern University of Science and Technology, Shenzhen 518055, China; Academy for Advanced Interdisciplinary Studies, Southern University of Science and Technology, Shenzhen 518055, China; Computational Bioscience Research Center (CBRC), Computer, Electrical and Mathematical Sciences and Engineering Division, King Abdullah University of Science and Technology (KAUST), Thuwal, Saudi Arabia; Shenzhen Key Laboratory of Gene Regulation and Systems Biology, Department of Biology, School of Life Sciences, Southern University of Science and Technology, Shenzhen 518055, China; Academy for Advanced Interdisciplinary Studies, Southern University of Science and Technology, Shenzhen 518055, China; Shenzhen Key Laboratory of Gene Regulation and Systems Biology, Department of Biology, School of Life Sciences, Southern University of Science and Technology, Shenzhen 518055, China; Academy for Advanced Interdisciplinary Studies, Southern University of Science and Technology, Shenzhen 518055, China

## Abstract

Alternative polyadenylation (APA) plays an important role in gene regulation. With the recent application of novel sequencing technology in APA profiling, an ever-increasing number of APA genes/sites have been identified. However, the phenotypic relevance of most of these APA isoforms remains elusive, which is largely due to the lack of a convenient genetics tool for APA interference. To address this issue, herein, an efficient method is developed based on the CRISPR-dCas13 system, termed as CRISPR-iPAS. Out of eight different dCas13 proteins, *Porphyromonas gulae* (Pgu) dCas13b, is identified as the most effective one in blocking the usage of the polyadenylation site (PAS). With guide RNAs targeting at core regulatory elements, dPguCas13b enabled APA regulation of endogenous genes with different APA types, including tandem 3′UTR, alternative terminal exon, as well as intronic PAS. Finally, we demonstrated that the proposed APA perturbation tool could be used to investigate the functional relevance of APA isoforms.

## INTRODUCTION

When polymerase II (Pol II) transcription reaches the end, nascent RNA is cleaved at its 3′ ends, followed by the addition of a poly(A) tail by a poly(A) polymerase (PAP). The coupled 3′ end cleavage and polyadenylation process (referred as polyadenylation hereafter) is not only essential for the maturation of most mRNAs, but also serves as an important mechanism in regulating their nuclear export, stability and translation (1,2). Polyadenylation is mediated by multiple protein complexes assembled at *cis*-elements around the 3′ end region of the pre-mRNA, including cleavage and polyadenylation specificity factors (CPSF), cleavage stimulation factor (CstF), cleavage factor I/II (CFI/IIm) and PAP (3–5). In mammals, the PAS signal, the most essential *cis*-element, is defined by the canonical AAUAAA hexamer and its close variants located 15–30 nucleotides upstream of the cleavage sites, which are recognized by the CPSF subunits: CPSF30 and Wdr33 (6,7). Other core elements include upstream UGUA element (USE) bound by cleavage factor Im (CFIm) and downstream U-rich or GU-rich elements (DSE) targeted by cleavage stimulation factor (CstF) (4,8,9).

In mammals, >70% of protein-coding genes express multiple transcript isoforms with distinct 3′ ends (10,11). The usage of these different polyadenylation sites (PAS) in the same gene is termed as alternative polyadenylation (APA). Depending on the location of the alternative PASs, APA events can be classified into different classes. The most common one is that all APA sites are located on the 3′ UTR, which is referred as ‘tandem 3′ UTR APA’. It produces transcripts with identical protein-coding sequences, but distinct 3′ UTRs of different lengths. These APA isoforms may contain different sets of *cis*-regulatory elements in their 3′ UTRs and therefore may differ in their mRNA stability (12,13), translation efficiency (14–16), intracellular localization (17,18), and even localization of encoded proteins (19). In addition to tandem 3′ UTR, there are three other types of APA events, including alternative terminal exon, intronic APA and internal exon APA (20–22). These APAs result in alternative isoforms that differ in their last exon, affecting not only 3′ UTR, but also the protein coding sequences, therefore, contributing also to the diversification of proteome repertoire (21,22). APA regulation is involved in a variety of biological processes, including cell proliferation (13,23), differentiation (24) and development (25,26), and its deregulation could cause several human genetic diseases as well as cancers (27,28).

With the recent application of next-generation sequencing technology in APA profiling, an ever-increasing number of APA genes/sites have been identified. Currently, PolyA_DB (as of August 2018), the most comprehensive APA database, annotated 290 168 PASs within 19 359 human genes (29). However, compared to the ubiquity of the phenomenon, studies, which clearly demonstrate the functions of different APA isoforms, are scarce, and the phenotypic relevance of most APA observed in high-throughput sequencing experiments remains elusive (30). The rather limited functional study is largely due to the lack of convenient genetic tools for APA perturbation.

Cas13 is a recently identified class 2 type VI CRISPR-Cas system, consisting of four family members, Cas13a, Cas13b, Cas13c and Cas13d. With convenient RNA-guiding and RNA-targeting capabilities, the CRISPR-Cas13 system has recently been applied for targeted RNA regulation (31–34). For instance, fused with ADAR2, nuclease-dead Cas13b (dCas13b) enabled the targeted adenine-to-inosine (A-to-I) editing, which has been further extended to targeted N6-methyladenosine (m^6^A) modification (35). Besides, dCas13d has also been used to modulate the mRNA splicing pattern by binding to splice sites, and thereby blocking its access by splicing machinery (36). More recently, dCas13b was engineered for targeted RNA imaging with high efficiency and specificity (37). Although the use of dCas13 has been successfully demonstrated in these different applications, it remains unclear whether dCas13 could be used for regulating polyadenylation and if yes, which family member would be the most efficient one.

In this study, we aimed to develop a convenient method based on dCas13 proteins, which can efficiently repress the usage of PASs with different features. For this purpose, using an APA reporter system, we first screened a panel of eight different dCas13 proteins and identified *Porphyromonas gulae* (Pgu) dCas13b as the most effective one in blocking the PAS usage. Then, we showed, by designing guide RNAs (gRNAs) to target core regulatory elements, the dCas13 system could be applied for regulating different APA types of endogenous genes, including tandem 3′UTR, alternative terminal exon as well as intronic PAS. Finally, we demonstrated that our APA perturbation tool could be used to investigate the functional relevance of APA isoforms.

## MATERIALS AND METHODS

### Cell culture and transfection

The HEK293T cells were obtained from the ATCC and cultured in DMEM (Gibco, C11965500BT) with 10% FBS (Gibco, 10270106) and 1% P/S (Gibco, 15070063) with 5% CO_2_ at 37°C.

For perturbation of PAS usage, HEK293T cells were seeded in 24-well plate and transfected at 70% cell confluence. For transfection, 3 μl PEI (1 μg/μl, Polysciences) was applied with 1 μg total plasmid (0.5 μg dCas13 and 0.5 μg individual gRNA). Forty-eight hours after transfection, cells were harvest for the following assays.

### Plasmid construction and establishment of the stable cell line

**Figure 1.**
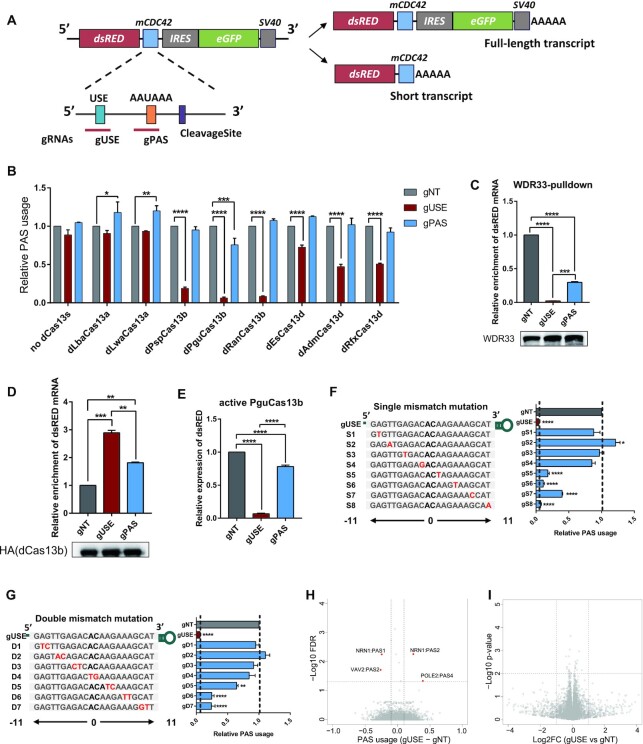
Repression of PAS usage with different dCas13 proteins. (**A**) Illustration of the reporter gene used to examine APA regulation. A 232-nucleotide (nt) sequence flanking an endogenous PAS from mouse gene *cdc42* was inserted. dsRED, red fluorescent protein; IRES, internal ribosome entry site; EGFP, enhance green fluorescent protein; AAAAA, poly(A) tail. (**B**) Relative usage of the target PAS in HEK293T^APA^ reporter cells transfected by one non-targeted control gRNA (gNT) and two gRNAs targeting at the PAS signal (gPAS) or USE (gUSE), respectively, together with or without different dCas13 proteins. Relative PAS usage was calculated by the abundance ratio between the two regions (dsRED/EGFP) measured using RT-qPCR and normalized to non-targeted gRNA (gNT). Without dCas13 proteins, both gRNAs showed no effect on the PAS usage, while six out of eight dCas13 proteins, dPspCas13b, dPguCas13b, dRanCas13b, dEsCas13d, dAdmCas13d and dRfxCas13d significantly reduced the PAS usage, when co-transfected with the USE-targeting gRNA (gUSE). Among the six effective dCas13 proteins, dPguCas13b showed the highest efficiency. Compared to gUSE, gRNA targeting PAS signal (gPAS) showed a much milder inhibitory effect. (**C**) RIP assays against WDR33 were performed in HEK293T^APA^ reporter cells with dPguCas13b and indicated gRNAs co-transfected, and the enrichment of dsRED mRNA was measured using RT-qPCR and normalized to the input total mRNA. The targeted reporter mRNA bound by WDR33 was significantly decreased in cells transfected with dPguCas13b and gUSE, compared to that in cells transfected with non-targeting control gRNA (gNT). Compared with gUSE, the decrease of WDR33 bound reporter mRNA was much subtler in cells transfected with gPAS. The immune-precipitated WDR33 protein in each RIP experiment was measured by Western Blot, as indicated in the lower panel. (**D**) RIP assays against HA-tagged dPguCas13b were performed in HEK293T^APA^ reporter cells co-transfected with HA-tagged dPguCas13b and indicated gRNAs, and the enrichment of dsRED mRNA was measured using RT-qPCR and normalized to the input mRNA. The targeted reporter mRNA bound by dPguCas13b was significantly higher in cells transfected with dPguCas13b and gUSE, compared to that in cells transfected with non-targeting control gRNA (gNT). Compared with gUSE, the enrichment of dPguCas13b bound reporter mRNA was significantly lower in cells transfected with gPAS. The protein level of dPguCas13b used in each RIP experiment was measured by Western Blot, as indicated in the lower panel. (**E**) Abundance of the reporter gene mRNA in HEK293T^APA^ reporter cells transfected with active PguCas13b and indicated gRNAs. The level of dsRED mRNA was measured using RT-qPCR and normalized to that of GAPDH. The knock-down effect induced by PguCas13b/gPAS was significantly milder than that with PguCas13b/gUSE. (**F**, **G**) Schematic view of the single (F) or double (G) gRNA-RNA mismatch positions within gUSE (Left), and the effects (Right) of corresponding mismatches on dPguCas13b PAS perturbation efficiency. Except the one at the very 3′ end, all the other single mismatches significantly impaired the repressive effect of dPguCas13b/gUSE complex. Among these mutants, mismatches at 5′ region of gRNA more significantly attenuated the interference on PAS usage than those at 3′ region (F). A stronger effect could be observed for double mismatches on the gRNA sequence, with a similar positional bias (G). B-G: For each experiment, three independent repeats were performed. Error bars represent SEM. *P < 0.05, **P < 0.01, ***P < 0.001, ****P < 0.0001, paired two-way Student's *t*-test. (**H**) The usage of endogenous PASs was measured by 3′ end mRNA sequencing in HEK293T^APA^ reporter cells co-transfected with dPguCas13b and gUSE or gNT. Out of 16,692 expressed PASs, only four PASs showed differential usage (FDR < 0.05 and difference of PAS usage > 0.1) between the cells transfected with gUSE and gNT. (**I**) The mRNA abundance of endogenous genes was measured by RNA-seq in HEK293T^APA^ reporter cells co-transfected with dPguCas13b and gUSE or gNT. Out of 14,812 expressed genes, no differential gene expression (*P*-value < 0.01 and log_2_ fold change > 1) between the cells transfected with gUSE and gNT was observed. H-I: For 3′ end mRNA sequencing, and RNA-seq, two replicates were performed for each experiment.

APA reporter was constructed by inserting a 232 nt region flanking the PAS of the mouse *cdc42* gene (Chr4: 137046876–137047107) into the pRiG vector (38). To establish the stable cell line, the whole APA-reporter region (Figure 1A) was then cloned into piggyBac cargo plasmid. After co-transfected with piggyBac cargo and transposase for 7 days, HEK293T cells with the medium expression level of dsRED fluorescence were sorted by flow cytometry.

The plasmids for different dCas13 proteins fused with 3xEGFP and their crRNA backbones were kindly provided by Lingling Chen's Lab (37). To establish the constructs expressing different dCas13 proteins without EGFP tagged, we cloned the coding sequence of each dCas13 with an HA at C-terminal and nucleus localization signal (NLS) at both terminals into the pHAGE-EF1α vector, using one-step clone kit (Vazyme). For gRNA expression, we designed 22 nt oligos to target core regulatory elements or non-targeted controls (NT) and inserted them into the crRNA vector by T4 ligation (37). The sequences of gRNAs were listed in Supplementary Table.

### RNA extraction and RT-qPCR

Total RNA was extracted using RNA Isolator Total RNA Extraction Reagent (Vazyme, R401-01). First-strand cDNA was synthesized using the HiScript III 1^st^ Strand cDNA Synthesis Kit (Vazyme, R312-02) with oligo dT as reverse transcription (RT) primer. Quantitative PCR (qPCR) was performed using Hieff qPCR SYBR Green Master Mix (Yeasen, 11201ES08), and was run on the BIO-RAD real-time PCR system. GAPDH was used as the control gene for normalization. The relative expression levels of target genes were normalized to those of GAPDH by using the ΔCt method. Primer sequences were listed in Supplementary Table. Three independent biological replications were performed for each experiment. The fold change of gene expression was calculated according to 2^ΔΔCt^.

### RNA immunoprecipitation (RIP)

Cells were harvested with NP40 lysis buffer (15 mM Tris–HCl pH 7.5, 350 mM NaCl, 0.1 mM EDTA, 1% NP-40 in DEPC H_2_O). For protein immunoprecipitation, protein A/G beads (MCE) were incubated with indicated antibody (1 μg for HA antibody and 0.5 μg for WDR33 per sample) in PBS at 4°C for 1 h. After two washes with NP40 lysis buffer, antibody-incubated beads were then incubated with cell lysate at 4°C for 2 h. After wash three times with lysis buffer, RNA isolator (Vazyme) was used to isolate RNA from beads. The abundance of enriched mRNA was measured by qPCR and normalized to indicated input mRNA.

### Western blotting

To measure the protein level, cells were collected and lysed by RIPA buffer (150 mM NaCl, 50 mM Tris, 1% EDTA, 1% NP40, 0.1% SDS), and centrifuged for 10 minutes at 12 000 rpm at 4°C. The total protein concentration was measured by BCA (Beyotime, P0011). Thirty micrograms of total protein were loaded and separated on the 10% SDS-polyacrylamide gradient gel. The proteins were then transferred onto polyvinylidene difluoride membranes (Immobilon-P, IPVH00010) and blocked with 5% non-fat milk (BBI, A600669) for 1 h at room temperature. The membranes were incubated with primary antibody and horseradish peroxidase-conjugated secondary antibody, and proteins were then detected using the Pierce™ ECL Western Blotting Substrate (Thermo, 32209) on BIO-RAD ChemiDoc™ XRS+ system, and the data were quantified by ImageJ. IGF2BP1 antibody (Proteintech) were used at a dilution of 1:1000, and GAPDH (Proteintech) was used as the control gene for normalization. Goat-anti-mouse antibody (TRANSGEN, HS201) was used as a secondary antibody at a dilution of 1:3000.

### 3′ RACE

To determine the polyadenylation site, cDNA was first synthesized using anchored oligo dT as RT primer. cDNA was then amplified by nest PCR using gene specific forward primers and anchor reverse primer. PCR products were separated by agarose gel and purified by Gel DNA Exaction mini kit (Vazyme). Purified PCR products were sent for Sanger sequencing and visualized by SnapGene Viewer. Primer sequences for 3′RACE assay were listed in Supplementary Table.

### mRNA stability measurement

Cells were treated with Actinomycin D (10 μg/ml) and collected after 0, 2, 4 and 8 h. Total RNA was isolated, and the mRNA abundance was estimated by using RT-qPCR. The half-life of mRNA was calculated as described by Huang *et al.* (39). In brief, assuming that mRNA decay follows first-order kinetics, the change of mRNA concentration at a given time (d*C/*d*t*) is proportional to the rate constant for decay (−k_decay_) and the concentration of remaining mRNA (*C*):

\begin{equation*}{\mathrm{\ }}{\rm d}C/d{\rm }t = - {k_{{\rm decay}}}\ *C\end{equation*}

As a result, the decay rate could be calculated as:

\begin{equation*}{\rm Ln}\ \left( {C/{C_0}} \right) = - {k_{{\rm decay}}}\ *t\end{equation*}

where C_0_ is the mRNA concentration at the starting time.

Then the mRNA half-life (*t*_1/2_) could be determined by:

\begin{equation*}{\rm Ln} \left( {1/2} \right) = \ - {k_{{\rm decay}}}*{t_{1/2}}\end{equation*}

So where:

\begin{equation*}{\mathrm{\ }}{t_{1/2}} = \ {\rm ln}2/{k_{{\rm decay}}}\end{equation*}

### Polysome profiling

Cells were first grown to 80% confluency. Prior to lysis, cycloheximide (100 μg/ml) was added into cells at 37°C for 10 min to halt the translating ribosomes. Cells were then washed with ice-cold PBS (supplemented with 100 μg/ml cycloheximide) and lysed in 300 μl of ice-cold lysis buffer (10 mM HEPES pH 7.4, 150 mM KCl, 10 mM MgCl_2_, 1% NP40, 0.5 mM DTT, 100 μg/ml cycloheximide, 1× proteinase inhibitor cocktail, 40 U/ml RNase inhibitor). After the cells were lysed by passing ten times through 25-gauge needle, the nuclei and the membrane debris were removed by centrifugation (15000 *g*, 10 min, 4°C). The supernatant (200 μl) was then layered onto a 10-ml linear sucrose gradient (10–50% [w/v], supplemented with 10 mM HEPES pH 7.4, 150 mM KCl, 10 mM MgCl_2_, 0.5 mM DTT, 100 μg/ml cycloheximide) and centrifuged (36 000 rpm, 140 minutes, 4°C) in an SW41Ti rotor (Beckman). After centrifugation, gradient containing layered fractions was read by Biocomp Gradient Fractionator, and fractions containing polysome (at least two ribosomes) RNAs were manually collected. Total mRNA of each fraction was isolated by Trizol-LS reagent (Yeason) and mRNA abundance was estimated by using RT-qPCR.

### Cell cycle and cell proliferation assay

For cell cycle assay, cells were harvested 72 h after post-transfection, and fixed with 70% ice cold ethanol at 4°C overnight. The fixed cells were then incubated with 200 ng/ml of RNase A at 37°C for 30 min and followed with 50 ug/ml of propidium iodide (PI) at room temperature for another 10 min. DNA content of cells were measured using a flow cytometer, and the percentage of cells in G1, S and G2 were analyzed by Flow Jo.

Cell proliferation rate was determined using Cell Counting Kit 8 (CCK8). For CCK8 assays, 1.5 × 10^4^ cells were seeded into 48-well plates. CCK8 solution (TargetMol) was added at indicated time points and then absorbance was measured at 450 nanometers using a microplate reader.

### Estimation of relative PAS usage

To estimate the relative usage of the proximal PAS within the tandem 3′ UTR APA, two qPCR primer pairs were designed targeting its upstream (UP) region and the region crossing the cleavage site (CVS), respectively. The relative PAS usage was calculated as the ratio between the two (UP/CVS) and normalized to samples transfected with non-targeting gRNA (NT). For the distal PAS within the tandem 3′UTR APA, one pair of qPCR primer was designed to estimate the abundance of full-length transcript by targeting the most distal region (FL) and the other targeting the upstream region of the proximal PAS. The relative PAS usage was calculated as the ratio of FL/UP. In the case of intronic PAS and alternative terminal exon PAS, two qPCR primer pairs were designed targeting its proximal (PE) and distal (DE) exon, respectively. Relative PAS usage was calculated as the ratio of PE/DE, and then normalized to samples transfected with non-targeting gRNA.

### 3′ end mRNA sequencing and data analysis

The library for 3′ end mRNA sequencing was prepared using QuantSeq 3′mRNA-Seq Library Prep Kit REV for Illumina (Lexogen). Samples were then sent for Illumina high-throughput sequencing.

To get clean reads from the raw 3′mRNA-seq data, adaptor sequences were firstly trimmed with cutadapt (40) and the leading T at the forward read (read 1) was further removed. Next, the clean forward reads were aligned to human reference genome (hg38) using STAR (41) with default parameters. The reference PAS with precise cleavage positions determined by 3′READS method (42) was obtained from PolyA_DB3 (29). For each PAS in the reference, the uniquely mapped reads with their 5′ end within a distance of 24 nt were counted using featureCounts (43). PASs with at least five reads in more than one samples were kept and DEXSeq (44) was applied to quantify the PAS usage difference across samples. For genes with multiple PASs, usage of each PAS (PAU) was calculated as its read count divided by the total read counts of all the PASs from the same gene.

### mRNA sequencing and data analysis

The library for mRNA sequencing was generated using Hieff NGS MaxUp II Dual-mode mRNA Library Prep Kit for Illumina (Yeasen). To analyze the sequencing data, we applied cutadapt (40) to remove the adaptor sequence from raw mRNA sequencing data and the trimmed reads were aligned to human reference genome (hg38) using STAR (41) with default parameters. Gene annotation was obtained from Gencode (v37) and the uniquely mapped reads in each gene were calculated by featureCounts (43). DESeq2 (44) was applied to compare gene expression across different samples.

## RESULTS

### dPguCas13b showed the highest efficiency in repressing the PAS usage on an APA reporter

To check if dCas13 proteins could be used to perturb PAS usage and, if yes, which member would be the most efficient one, we first screened a panel of nuclease-dead Cas13 (dCas13) proteins, including *Lachno-spiraceae bacterium* (Lba) dCas13a, *Leptotrichia wadei* (Lwa) dCas13a, *Prevotella* sp. *P5-125* (Psp) dCas13b, *Porphyromonas gulae* (Pgu) dCas13b, *Riemerella anatipestifer* (Ran) dCas13b, *Eubac-terium siraeum DSM15702* (Es) dCas13d, *Anaerobic digester metagenome 15706* (Adm) dCas13d and *Ruminoccocus flavefa-ciensXPD3002* (Rfx) dCas13d (32,33,36,45,46). For this purpose, we used an APA reporter based on the pRiG vector, which can generate two transcript variants when a proximal PAS is inserted (Figure 1A) (47,48). The higher usage of the proximal PAS would result in the higher relative abundance of the short transcript. Therefore, we could estimate the relative usage of the cloned PAS based on the ratio of short versus full-length transcript.

As shown in Figure 1A, to generate the proximal PAS, we inserted in the reporter a 232-nucleotide (nt) sequence flanking the endogenous PAS of mouse *cdc42* (Figure 1A, see also Materials and Methods). This region contains an AAUAAA as the PAS signal, and an upstream UGUA motif (USE). To better mimic APA regulation in cells, we established a HEK293T^APA^ cell line stably expressing the APA reporter at relative medium level (see Method). Since the core regulatory elements around PAS are essential for the polyadenylation process, intuitively, blocking the access of the polyadenylation machinery to these elements would be an effective way to repress the PAS usage. Therefore, we designed two gRNAs, targeting at the AAUAAA PAS signal (gPAS) and the upstream element (gUSE) of the inserted PAS, respectively. Two gRNAs were transfected separately into HEK293T cells with or without different dCas13 proteins. As shown in Figure 1B, without dCas13 proteins, both gRNAs showed no effect on the PAS usage, while six out of eight dCas13 proteins, dPspCas13b, dPguCas13b, dRanCas13b, dEsCas13d, dAdmCas13d and dRfxCas13d significantly reduced the PAS usage, when co-transfected with the USE-targeting gRNA (gUSE). Among the six effective dCas13 proteins, dPguCas13b showed the highest efficiency, followed by dRanCas13b, dPspCas13b, dRfxCas13d, dAdmCas13d and dEsCas13b (Figure 1B). Therefore, we selected dPguCas13b for all the following experiments.

We hypothesized that the observed repressive effect was caused by the competitive binding between dCas13 protein and polyadenylation machinery at the gRNA-targeted PAS regulatory elements. To check whether it is the case, we pulled down the RNA bound with WDR33, a member of CPSF complex recognizing the PAS signal (6), in cells co-transfected with dPguCas13b and targeting (gUSE) or non-targeting gRNA (gNT), respectively. As shown in Figure 1C, the targeted reporter mRNA bound by WDR33 was significantly decreased in cells transfected with dPguCas13b and gUSE, compared to that in cells transfected with non-targeting control gRNA (gNT). This validated our hypothesis that dPguCas13b with indicated gRNAs disrupted the binding of WDR33 to the targeted PAS site, thereby repressed its usage.

Interestingly, as shown in Figure 1B, gRNA targeting AAUAAA (gPAS) showed a much milder inhibitory effect than gUSE (Figure 1B). To figure out the potential cause, we compared the target sequences of the two gRNAs and found that the sequence targeted by gPAS is of a very low GC content (27%). To check whether this could result in a low binding affinity of dCas13/gPAS complex to its target, we pulled down the RNA bound with dPguCas13b in cells transfected with dPguCas13b and gPAS, gUSE or non-targeting gRNA (gNT), respectively. As shown in Figure 1D, although compared with gNT, dPguCas13b complexed with gPAS could significantly enrich target reporter RNA, the abundance of targeted reporter RNA bound to dPguCas13b was significantly lower when it was complexed with gPAS than with gUSE. In consistent with the observed lower binding of target site by dPguCas13b/gPAS, the abundance of targeted reporter mRNA bound by WDR33 was significantly higher in cells transfected with dPguCas13b/gPAS than that with dPguCas13b/gUSE (Figure 1C). To further validate the functional relevance of the observed lower targeting efficiency of gPAS, we transfected active PguCas13b with these two gRNAs respectively and compared their efficiency in knocking down the target reporter RNA. As shown in Figure 1E, the effect induced by PguCas13b/gPAS was significantly milder than that with PguCas13b/gUSE. Taken together, we demonstrated that dPguCas13b, together with gRNAs targeting core regulatory elements of polyadenylation, enabled the efficient repression of the targeted PAS, and the binding affinity of gRNA to its target was a critical factor to be considered in gRNA design.

### dPguCas13b could achieve a high specificity in APA interference

Off-target effect is a critical issue for all application of CRISPR/Cas-based systems. To address this issue, we assessed the target specificity of our dCas13-based PAS perturbation tool with two strategies. Given that the specificity was mainly determined by the base-pairing between gRNA and target site, we first evaluated the importance of number and position of the mismatch in the target/gRNA duplex. For this purpose, either single or double mismatches were systematically introduced across the gRNA sequence, and the efficiency in repressing the PAS usage was then measured for each mutated gRNA separately. As shown in Figure 1F, except the one at the very 3′ end, all the other single mismatches significantly impaired the repressive effect of dPguCas13b/gUSE complex. Among these mutants, mismatches at 5′ region of gRNA more significantly attenuated the interference on PAS usage than those at 3′ region (Figure 1F), which was also observed for dPspCas13b mediated RNA targeting (37). As expected, a stronger effect could be observed for double mismatches on the gRNA sequence, with a similar positional bias (Figure 1G). Second, to evaluate the specificity of dPguCas13b for APA interference in a genome-wide manner, we measured the usage of all the endogenous PAS in HEK293^APA^ cells co-transfected with dPguCas13b and gUSE or control non-targeting gRNA. Whereas the significantly decreased usage of the reporter PAS was validated (Supplementary Figure S1), out of 16 692 endogenously expressed PASs, only four PASs showed differential usage between the cells transfected with gUSE and those with control non-targeting gRNA (Figure 1H). However, none of these four PASs contained the sequences that base-pair with gUSE with at most two mismatches in their 100 nt flanking regions. In addition, we also performed RNA-seq in HEK293^APA^ cells co-transfected with dPguCas13b and gUSE or control non-targeting gRNA. As shown in Figure 1I, out of 14 812 endogenously expressed genes, no significant differentially expressed gene was observed between the cells transfected with gUSE and those with control non-targeting gRNA. Therefore, our data from the systematic mutagenesis of gRNA sequence as well as global measurement of PAS usage and gene expression demonstrated dPguCas13b could achieve a high specificity based on gRNA-target RNA base-pairing.

### CRISPR-iPAS enabled the repression of endogenous PAS usage

With the successful application of dPguCas13b in repressing the proximal PAS in the APA reporter, we turned to the endogenous candidate PASs of different APA types, including tandem 3′UTR, intronic APA, and alternative terminal exon APA. For this purpose, we estimated the usage of PASs expressed in HEK293T cells and chose the PAS with dominant usage for the following APA perturbation.

**Figure 2.**
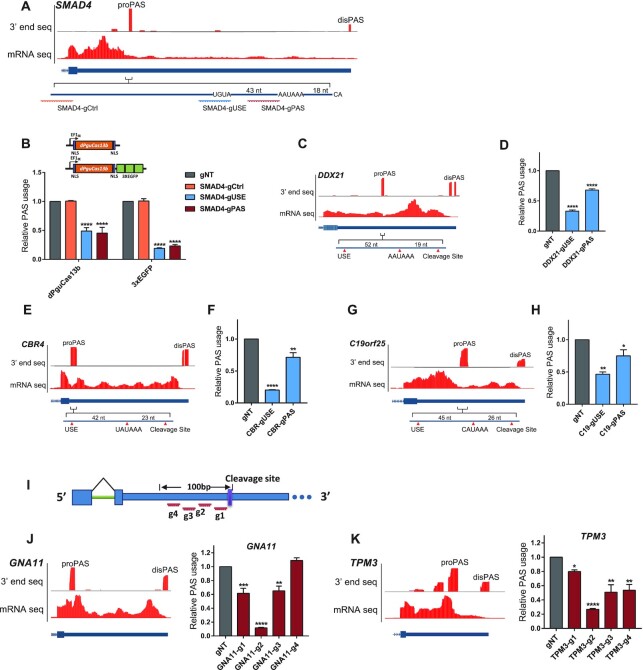
dPguCas13b inhibited endogenous tandem 3′ UTR APA. (**A**) UCSC genome browser tracks for RNA-Seq and 3′end mRNA Seq were shown for the last exon of the human gene SMAD4. The proximal PAS with the dominant usage (proPAS) and the distal PAS (disPAS) were indicated. The positions of primer target regions and gRNA target regions were shown along with the proPAS. (**B**) The PAS usage was repressed by dPguCas13b or 3xEGFP-dPguCas13b co-transfected with gRNA targeting AUUAAA PAS signal (SMAD4-gPAS) and UGUA (SMAD4-gUSE) of SMAD4, but not with gRNAs targeting the sequence outside of the core PAS regulatory region (SMAD4-gCtrl). Compared with dPguCas13b alone, the reduction of PAS usage was increased after 3xEGFP fusion. (C-H) Perturbation of the dominant PAS expressed in human gene DDX21, CBR4 and C19orf25. UCSC genome browser tracks for RNA-Seq and 3′end mRNA Seq were shown for the last exon of the human gene DDX21 (**C**), CBR4 (**E**) and C19orf25 (**G**). The proximal PAS with the dominant usage (proPAS) and the distal PAS (disPAS) were indicated. The PAS usage was repressed by 3xEGFP-dPguCas13b co-transfected with gRNA targeting PAS signal (gPAS) and UGUA (gUSE) of gene DDX21 (**D**), CBR4 (**F**) and C19orf25 (**H**), respectively. (**I**) Four gRNAs were designed to target the 100 nt region upstream of the PAS cleavage sites in the 3′UTRs of gene GNA11 and TPM3. (J-K) UCSC genome browser tracks for RNA-Seq and 3′end mRNA Seq was shown for the last exon of human gene GNA11 (**J**) and TPM3 (**K**) in the left panel. The proximal PAS with the dominant usage (proPAS) and the distal PAS (disPAS) were indicated. The relative PAS usage in gene GNA11 (J) and TPM3 (K) in HEK293T cells after transfection of 3xEGFP-dPguCas13b together with indicated gRNAs were shown in the right panel. A gRNA targeting on the region 40–61 nt (GNA11-g2) or 30–51 nt (TPM3-g2) upstream of the cleavage site induced the strongest inhibitory effect on the PAS usage. For each experiment, three independent repeats were performed. Error bars represent SEM. *P < 0.05, **P < 0.01, ***P < 0.001, ****P < 0.0001, paired two-way Student's *t*-test.

Tandem 3′UTR APA is the most common APA form, which leads to mRNA isoforms with distinct 3′UTR of different lengths. For example, as shown in Figure 2A, SMAD4 expressed six PASs along its 3′ UTR, among which, the second proximal one was of the highest usage in HEK293T cells (Figure 2B). To repress the PAS usage, we designed two gRNAs, targeting at the AAUAAA PAS signal (SMAD4-gPAS) and USE (SMAD4-gUSE), respectively (Figure 2A). To estimate the relative usage of the dominant PAS, two pairs of qPCR primers were designed to target its upstream region (UP) and the region crossing the cleavage site (CVS), respectively, and the ratio of their abundance was used as a proxy for the relative PAS usage. As shown in Figure 2B, both gRNAs could induce a significant decrease in PAS usage. To check whether such repressive effect could only be achieved by specifically targeting core regulatory elements, we designed another gRNA targeting the sequence outside of the core PAS regulatory region (SMAD4-gCtrl). As expected, similar as the non-targeting gNT, this SMAD4-gCtrl could not perturb the usage of the dominant PAS (Figure 2B).

Mechanistically, our dCas13-based system inhibited the polyadenylation process via interfering the access to the core elements by polyadenylation machinery (Figure 1C). Therefore, intuitively, increasing the steric hinderance by dCas13 protein might enhance the inhibitory effect. To test this hypothesis, we fused dPguCas13b with 3xEGFPs (Figure 2B, Materials and Methods). As expected, 3xEGFP-fused dPguCas13b exhibited more efficient repression on the PAS usage with both gRNAs. Compared with dPguCas13b alone, the reduction of PAS usage was significantly increased after 3xEGFP fusion (Figure 2B). Similar inhibitory effect by EGFP-fused dPguCas13b was detected in another proximal PAS dominantly used in gene *DDX21* (Figure 2C, 2D). Taken together, 3xEGFP-fused dPguCas13b allowed more efficient perturbation of polyadenylation and was therefore used in all the following studies, referred hereafter as CRISPR-iPAS.

Except for the canonical AAUAAA, several other hexamer variants can also be used as PAS signals, but with lower strengths (49,50). Therefore, we further checked the CRISPR-iPAS on PASs with other poly(A) signal variants. In *CBR4*, a gene encoding a Carbonyl Reductase 4, two PASs are located on its 3′ UTR, of which the dominant one uses UAUAAA as its PAS signal (Figure 2E). As shown in Figure 2F, dPguCas13b with the gRNAs targeting the USE or PAS signal could significantly block the PAS usage. A similar repressive effect could also be achieved in a PAS located in the 3′ UTR of gene *C19orf25*, which uses CAUAAA as its PAS signal (Figure 2G and H). These results demonstrated that CRISPR-iPAS can block the PAS usage by targeting the PAS signal with different hexamer variants.

There are about 10% of PASs without known PAS signal variants upstream of their cleavage sites (51). For example, *GNA11*, a gene encoding G protein subunit alpha 11, expressed two PASs located in the 3′UTR, of which the one with the higher usage contained no known PAS signal variant (Figure 2J). To check whether CRISPR-iPAS can also perturb polyadenylation of such PAS, we designed four gRNAs with target sites across the 100 nt region upstream of its cleavage site (Figure 2I). As shown in Figure 2J, GNA11-gRNA2 targeting at the region 40–61 nt upstream of the cleavage site showed the highest inhibitory effect, whereas GNA11-gRNA1 and GNA11-gRNA3 could only induce much subtle repression. A similar effect could also be achieved for the PAS of the same type in the 3′ UTR of gene *TPM3*, where TPM3-gRNA2 targeting at the region 30–51 nt upstream of the cleavage site could significantly decrease the PAS usage (Figure 2K). These results demonstrated that CRISPR-iPAS can be used to inhibit the usage of the PASs even without a known PAS signal. Interestingly, for both PASs tested here, the gRNAs with the highest efficiency were targeted on the region, where known PAS signal variants were located. This indicates that CPSF complex may bind in the same region for all the PASs with or without known hexamer variants.

In addition to tandem 3′ UTR APA, alternative PASs located upstream of the last exon could lead to transcript isoforms that differed in both coding and 3′ UTR sequences. Such APA could be classified into three groups: alternative terminal exon APA, intronic APA and internal exon APA. Internal exon APA often results in transcripts without an in-frame stop codon, which are degraded rapidly through the non-stop decay pathway, and is relatively rare (52). Therefore, we focused here only on intronic or alternative terminal exon APA.

**Figure 3.**
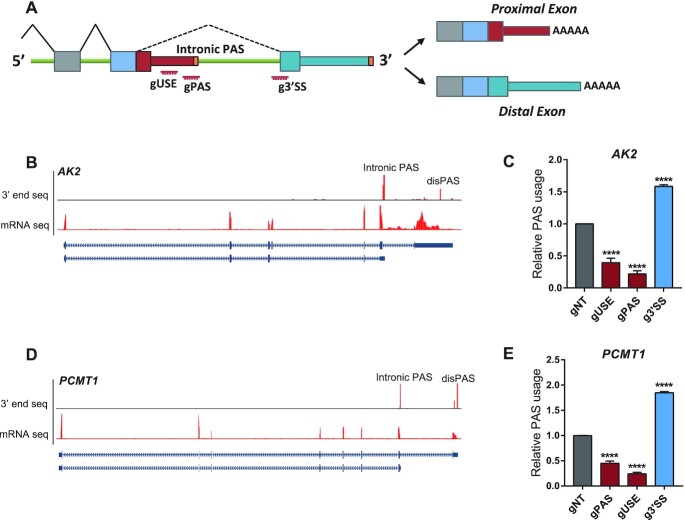
dPguCas13b enabled both repression and enhancement of the intronic PAS usage. (**A**) Illustration of genes with intronic APA. The positions of gRNA target regions were indicated. (**B**) UCSC genome browser tracks for RNA-Seq and 3′end mRNA Seq were shown for the human gene AK2. The intronic PAS with the dominant usage (Intronic PAS) and the distal PAS (disPAS) were indicated. (**C**) The usage of the intronic PAS in gene AK2 was repressed after transfection of 3xEGFP-dPguCas13b together with gRNAs targeting the PAS signal (gPAS) and USE (gUSE), but enhanced with gRNA targeting the 3′ splicing site (g3′SS). (**D**) UCSC genome browser tracks for RNA-Seq and 3′end mRNA Seq was shown for the human gene PCMT1. The intronic PAS with the dominant usage (Intronic PAS) and the distal PAS (disPAS) were indicated. (**E**) The usage of the intronic PAS in gene PCMT1 was repressed after transfection of 3xEGFP-dPguCas13b together with gRNAs targeting the PAS signal (gPAS) and USE (gUSE), but enhanced with gRNA targeting the 3′ splicing site (g3′SS). For each experiment, three independent repeats were performed. Error bars represent SEM. ****P < 0.0001, paired two-way Student's *t*-test.

Intronic polyadenylation (IPA) ends the mRNA within an otherwise intron and extends the preceding internal exon as the terminal exon (Figure 3A). A recent study has reported a widespread upregulation of IPA in chronic lymphocytic leukemia (CLL) patients where the IPA-generated transcripts often lack the region encoding tumor-suppressive domain (53). Mechanistically, the usage of IPA is the net result of competition between splicing and polyadenylation. Therefore, whereas inhibition of polyadenylation would repress IPA, that of splicing could enhance IPA (54–56). To check whether CRISPR-iPAS could be used for both IPA inhibition and enhancement, we tested it on two IPAs. As shown in Figure 3B, *AK2*, a gene encoding the phosphotransferase adenylate kinase 2, expressed an intronic PAS upstream of the last exon. To estimate the relative usage of the intronic PAS, we designed two primer pairs targeting on proximal exon (PE) and distal exon (DE), respectively, and the ratio of their abundance was used as a proxy for the relative PAS usage (Figure 3A). To inhibit the PAS usage, similar as before, we designed two gRNAs targeting on PAS signal and USEs, respectively. As shown in Figure 3C, the two gRNAs could significantly reduce the PAS usage. To enhance the PAS usage, we designed one gRNA targeting at the 3′ splicing site (g3′ SS), based on the hypothesis that binding of dCas13 might interfere with the access of U2-snRNP. Indeed, the 3′SS targeting could significantly enhance the usage of intronic PAS (Figure 3C). A similar inhibitory/enhancing effect could be also achieved for another intronic PAS in gene *PCMT1* (Figure 3D and 3E).

**Figure 4.**
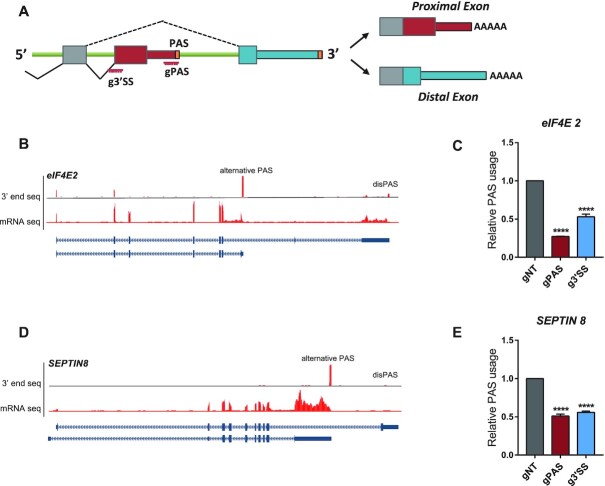
dPguCas13b repressed the usage of alternative terminal exon PAS. (**A**) Illustration of genes containing alternative terminal exon PASs. The positions of gRNA target regions were indicated. (**B**) UCSC genome browser tracks for RNA-Seq and 3′end mRNA Seq were shown for the human gene eIF4E2. The alternative terminal exon PAS with the dominant usage (alternative PAS) and the distal PAS (disPAS) were indicated. (**C**) The usage of the alternative PAS in gene eIF4E2 was repressed after transfection of 3xEGFP-dPguCas13b together with gRNAs targeting the PAS signal (gPAS) and 3′ splicing site (g3′SS). (**D**) UCSC genome browser tracks for RNA-Seq and 3′end mRNA Seq were shown for the human gene SEPTIN8. The alternative terminal exon PAS with the dominant usage (alternative PAS) and the distal PAS (disPAS) were indicated. (**E**) The usage of the alternative PAS in gene SEPTIN8was repressed after transfection of 3xEGFP-dPguCas13b together with gRNAs targeting the PAS signal (gPAS) and 3′ splicing site (g3′SS). For each experiment, three independent repeats were performed. Error bars represent SEM. ****P < 0.0001, paired two-way Student's *t*-test.

Alternative terminal exon APA is a special type of alternative splicing, caused by the mutually exclusive usage of the alternative 3′ SS (22) (Figure 4A). Therefore, the interference of both the splicing and polyadenylation process would likely perturb the PAS usage. As shown in Figure 4A, *eIF4E2* (eukaryotic initiation factor 4E 2) expressed an alternative terminal exon APA, located upstream of the last exon (Figure 4B). To investigate if CRISPR-iPAS could perturb its usage, we designed two gRNA targeting the PAS signal and the 3′ SS of the alternative exon, respectively. As expected, dPguCas13b blocked usage of the PAS together with gRNA targeting on the PAS signal and 3′ SS (Figure 4C). Similar effects were also observed for another alternative terminal exon PAS expressed in gene *SEPTIN8* (Figure 4D and 4E).

### CRISPR-iPAS exerted variable effect on the mRNA abundance

**Figure 5.**
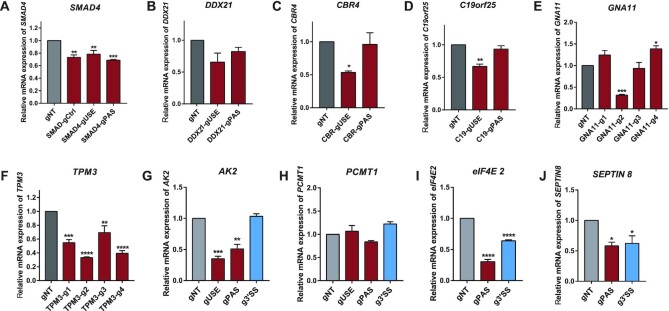
CRISPR-iPAS targeted at proximal PAS often exerted negative effect on the total mRNA abundance. The total mRNA abundance was measured in HEK293T cells with 3xEGFP-dPguCas13b and indicated gRNAs transfected for gene SMAD4 (**A**), DDX21 (**B**), CBR4 (**C**), C19orf25 (**D**), GNA11 (**E**), TPM3 (**F**), AK2 (**G**), PCMT1 (**H**), eIF4E2 (**I**) and SEPTIN8 (**J**). For the six genes with tandem 3′UTR APA, including SMAD4, DDX21, CBR4, C19orf25, GNA11 and TPM3, the total mRNA level was often significantly decreased when their proximal PAS was perturbed by CRISPR-iPAS (A–F). The decrease of total mRNA level induced by different gRNAs appeared to correlate with their inhibitory effects on the PAS usage. In the genes containing intronic APA, CRISPR-iPAS targeting the proximal PAS significantly reduced the total mRNA level of AK2 (**G**), but caused no effect on PCMT1 (**H**), whereas no effect was observed with CRISPR-IPAS targeting 3′SS on either AK2 or PCMT1 (G, H). For the genes with alternative-terminal-exon APA including SEPTIN8 (**I**) and eIF4E2 (**J**), CRISPR-IPAS targeting either PAS or 3′SS significantly decreased the total mRNA abundance.

As polyadenylation is essential for mRNA maturation, in addition to modulating the relative PAS usage, the interference of polyadenylation process might also reduce the abundance of total mRNA from the same gene. To investigate such effects, we measured the total mRNA abundance using primers targeting on the common coding region (CDS). As shown in Figure 5A–H, CRISPR-iPAS exerted variable effects on total mRNA abundance for different PASs with different gRNAs. For the six genes with tandem 3′UTR APA, including *SMAD4*, *DDX21*, *CBR4, C19orf25*, *GNA11* and *TPM3*, the total mRNA level was often decreased when their proximal PAS was perturbed by CRISPR-iPAS (Figure 5A–F). The decrease of total mRNA level induced by different gRNAs appeared to correlate with their inhibitory effects on the PAS usage (see also Figure 3). In the genes containing intronic APA, CRISPR-iPAS targeting the proximal PAS significantly reduced the total mRNA level of *AK2* (Figure 5G), but caused no effect on *PCMT1* (Figure 5H), whereas no effect was observed with CRISPR-iPAS targeting 3′SS on either *AK2* or *PCMT1* (Figure 5G, 5H). For the genes with alternative-terminal-exon APA including *SEPTIN8* (Figure 5I) and *eIF4E2* (Figure 5J), CRISPR-iPAS targeting either PAS or 3′SS decreased the total mRNA abundance. Taken together, perturbation of RNA processing including both polyadenylation and splicing could reduce the level of mature mRNA, although the effect could be variable. Interestingly, as shown in Figure 5A, the gRNA targeting the sequence outside of the core PAS regulatory region in gene *SMAD4* (SMAD4-gCtrl), which did not affect the PAS usage, also led to the reduction of mRNA abundance to a similar extent as the gUSE with strong effect on PAS usage. Similarly, the gRNA1 used in *TPM3* experiment (TPM3-g1) caused only slight decrease in PAS usage (Figure 2K), but induced dramatic reduction of its total mRNA abundance (Figure 5F). This suggested that at least some of the observed effects on total mRNA abundance might arise independently from PAS perturbation.

**Figure 6.**
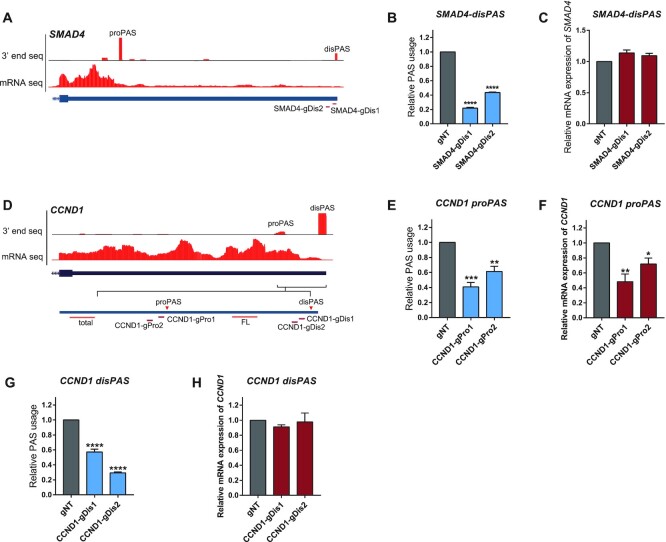
CRISPR-iPAS targeted at distal PAS exerted no negative effect on the mRNA abundance. (**A**) UCSC genome browser tracks for RNA-Seq and 3′end mRNA Seq were shown for the last exon of the human gene SMAD4. The position of gRNAs targeting the distal PAS (disPAS) were indicated. (**B**) The usage of the distal PAS in gene SMAD4 was repressed after transfection of 3xEGFP-dPguCas13b together with the two gRNAs targeting the PAS signal (gPAS). (**C**) None of the two gRNAs targeting the PAS signal (gPAS) caused significant negative effect on total mRNA abundance of SMAD4. (**D**) UCSC genome browser tracks for RNA-Seq and 3′end mRNA Seq was shown for the last exon of the human gene Cyclin D1 (CCND1). The proximal PAS (proPAS) and the distal PAS with the dominant usage (disPAS) were indicated. Two gRNAs were designed to target the PAS signal of the distal PAS and proximal PAS, respectively. (E–H) gRNAs targeting either (**E**) its proPAS or (**G**) disPAS could significantly repress the usage of their target PAS, whereas the gRNAs targeted at proximal PAS significantly decreased the total mRNA abundance (**F**), those targeted at distal PAS did not at all (**H**). For each experiment, three independent repeats were performed. Error bars represent SEM. *P < 0.05, **P < 0.01, ***P < 0.001, ****P < 0.0001, paired two-way Student's *t*-test.

Very recently, a study from Yu Zhou and Xiang-dong Fu's lab revealed that the isoforms ended with stronger distal PAS could undergo additional round of posttranscriptional cleavage at a proximal PASs to generate the short 3′UTR isoform (57). If so, the perturbation of the distal PAS might result in more efficient use of proximal PASs with little effect on total mRNA abundance. To check this possibility, we performed CRISPR-iPAS on distal PASs of SMAD4 by designing two gRNAs targeting at PAS signal of the distal PAS (Figure 6A). As shown in Figure 6B, CRISPR-iPAS targeting on the distal PAS of SMAD4 significantly blocked its usage. However, none of the two gRNAs caused significant negative effect on total mRNA abundance as observed for that on proximal PAS (Figure 6C). Given that in SMAD4 the proximal PAS was of a much higher usage than the distal one, it was plausible that the different effects on the total mRNA abundance observed for perturbation of the two PASs were due to their different usage. To address this, we chose *CCND1* (*CyclinD1*) with two PASs located in the 3′UTR, of which the distal one was of the dominant usage (Figure 6D). To perturb the distal and proximal PASs, we designed two gRNAs to target the PAS signal of either PAS, respectively (Figure 6D). As shown in Figure 6E and 6G, all the four gRNAs could significantly repress the usage of their targeted PAS. However, whereas the two gRNAs targeted at proximal PAS significantly decreased the total mRNA abundance (Figure 6F), those two targeted at distal PAS did not at all (Figure 6H). Taken together, these results suggested that whereas the perturbation of proximal PAS would interfere with the mRNA processing and therefore reduced the total mRNA abundance, that of distal PAS would likely convert the longer isoform to the short one without affecting its total mRNA level.

### CRISPR-iPAS enabled the functional investigation of APA isoforms

Due to the lack of convenient tools for APA perturbation, studies, where functions of different APA isoforms have been demonstrated, are still scarce. To demonstrate that CRISPR-iPAS can be used for the functional study of APA isoforms, as a proof-of-principle example, we chose *IGF2BP1*, a gene encoding Insulin-like growth factor 2 mRNA binding protein 1, and known to be involved in cellular proliferation, apoptosis, and invasion (58–60).

**Figure 7.**
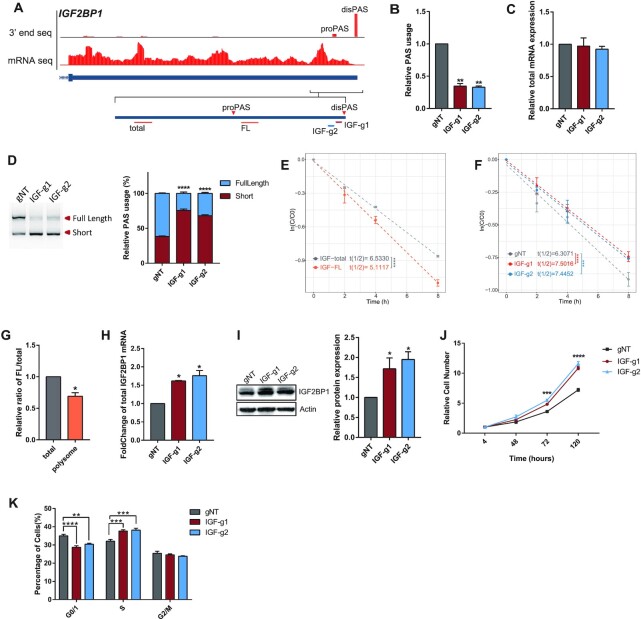
CRISPR-iPAS enabled the functional investigation of APA isoforms in gene IGF2BP1. (**A**) UCSC genome browser tracks for mRNA seq and 3′end mRNA Seq were shown for the last exon of human gene IGF2BP1. The proximal PAS (proPAS) and distal PAS with the dominant usage (disPAS) were indicated. The positions of primer target regions and gRNA target regions were shown along with the two PASs. (**B**) The usage of the distal PAS was repressed after transfection of 3xEGFP-dPguCas13b together with the two gRNAs targeting the PAS signal. (**C**) The total mRNA expression of IGF2BP1 in HEK293T cells co-transfected 3xEGFP-dPguCas13b together with gRNAs targeting the distal PAS did not show significant changes, compared to the non-targeting control gRNA (gNT). (**D**) 3′ RACE assay validated the switch of dominant PAS from the distal one to the proximal one after perturbation of the distal PAS. The cDNA products derived from APA isoforms ended with distal PAS (Full Length) and the proximal PAS (Short) were indicated and quantified by ImageJ (Right). (**E**) mRNA half-life of total (grey) and full-length (FL, orange) transcripts of IGF2BP1 were measured in unperturbed HEK293T cells. The half-life of IGF2BP1-FL was significantly shorter than that of the total IGF2BP1mRNA. (**F**) mRNA half-life of IGF2BP1 was increased after perturbation of distal PAS usage by CRISPR-iPAS with both gRNAs targeting at PAS signal (red and blue), compared to the gNT (gray). (**G**) The relative proportion of IGF2BP1-FL was lower in polysome fractions than in total cell lysate from unperturbed HEK293T cells. (**H**) CRISPR-iPAS perturbation of the distal PAS resulted in higher amount of IGF2BP1 mRNAs associated with polysome. (**I**) The protein level of IGF2BP1 was increased upon CRISPR-iPAS perturbation of the distal PAS. The protein level was measured by western blot (left panel) and quantified by ImageJ (right panel). Actin was used as loading control. (**J**) CCK-8 analysis was performed at 4, 48, 72 and 120 h after seeding the cells. The relative cell numbers in the indicated time points were shown for cells transfected with non-targeting control gRNA (Black) and gRNAs targeting at the PAS signal (Blue and Red). Cell proliferation rate was significantly increased after perturbation of the distal PAS. (K) Flow cytometry-based cell cycle analysis showed that the perturbation of distal PAS usage by CRISPR-iPAS increased and decreased percentage of cells in S and G0/G1 phase, respectively. For each experiment, three independent repeats were performed. Error bars represent SEM. *P < 0.05, ***P < 0.001, ****P < 0.0001, paired two-way Student's *t*-test.

As shown in Figure 7A, *IGF2BP1* mainly expressed two PASs located on its 3′ UTR in HEK293T cells, with the distal one being of the highest usage. To estimate the expression level of the two APA isoforms, we designed two pairs of qPCR primers: one was targeted at the region upstream of the proximal PAS and was used to measure the total expression of two isoforms together (total), while the other was targeted at the region located between the two PASs and was used to measure only the expression of the isoform ended with the distal PAS (FL) (Figure 7A). To repress the usage of the distal PAS, we designed two gRNAs targeted at its core region (IGF-g1, IGF-g2). As shown in Figure 7B, both gRNAs could induce significant repression of the distal PAS usage (Figure 7B), with only subtle effect on the total mRNA expression level (Figure 7C). 3′ RACE experiment further confirmed the changes in the relative abundance of the two APA isoforms upon CRISPR-iPAS perturbation (Figure 7D, Supplementary Figure S2A). As it is well-known that 3′UTR often contains *ci*s-elements regulating mRNA stability and/or translational efficiency, we further investigated whether the shortening of *IGF2BP1* 3′UTR could affect mRNA stability and translation. For this purpose, we first measured and compared the half-life between the full-length transcript (IGF2BP1-FL) and the total IGF2BP1 mRNA (Methods). As shown in Figure 7E, the half-life of IGF2BP1-FL was significantly shorter than the total IGF2BP1 mRNA, suggesting that the 3′UTR shortening might stabilize its mRNA. Indeed, the half-life of the total IGF2BP1mRNA was slightly prolonged upon CRISPR-iPAS perturbation of the distal PAS (Figure 7F). To check the effect on the translational efficiency, we performed polysome profiling first in unperturbed HEK293T cells and compared the relative proportion of IGF2BP1-FL isoform between polysome associated RNA and total RNA from cell lysates. As shown in Figure 7G, the relative proportion of IGF2BP1-FL was lower in polysome fractions (Figure 7G), suggesting the lower translation efficiency of the full-length isoform. Therefore, the 3′UTR shortening induced by inhibition of the distal PAS would lead to the enhanced translation and increased protein level of IGF2BP1. Indeed, CRISPR-iPAS perturbation of the distal PAS resulted in higher amount of IGF2BP1 mRNAs associated with polysome (Figure 7H), as well as a higher protein level of IGF2BP1 (Figure 7I). Therefore, our study demonstrated that the 3′ UTR shortening of *IGF2BP1* stabilized its mRNA and enhanced the translational efficiency, suggesting the presence of regulatory elements with the effect of RNA destabilizing as well as translational repression in the region between the two PASs. The identification of these elements warranted further study in the future.

It has been shown that upregulation of IGF2BP1 could accelerate cell proliferation via promoting G1-to-S transition (59,60). To investigate the effect of our CRISPR-iPAS perturbation on cell proliferation, we performed CCK-8 assay to measure and compare the proliferation rate of cells with or without PAS perturbation. In consistent with the positive effect of perturbation on IGF2BP1 protein level, cell proliferation rate was significantly increased after perturbation of the distal PAS (Figure 7J). To further investigate the underlying mechanism, we profiled the proportion of cells at different cell cycle stages using flow cytometry. As shown in Figure 7K, after CRISPR-iPAS perturbation, the proportion of cells in G0/G1 phase decreased, whereas that in S phase increased (Figure 7K, Supplementary Figure S2B). Taken together, using IGF2BP1 as an example, we demonstrated that CRISPR-iPAS could be used for mechanistic study of the APA function.

## DISCUSSION

In this study, we developed a convenient method for perturbation of PAS usage. The so termed ‘CRISPR-iPAS’ was based on 3xEGFP-fused dPguCas13b protein, which could be guided by gRNAs targeting at PAS core regulatory regions thereby blocking their access by polyadenylation machinery. CRISPR-iPAS could be applied for regulating different APA types with high efficiency and specificity, including tandem 3′UTR, alternative terminal exon as well as intronic PAS. Finally, using gene *IGF2BP1* as an example, we demonstrated that CRISPR-iPAS could be used for the mechanistic study of functional relevance of APA isoforms.

Similar as all the other CRISPR systems, the target specificity of CRISPR-iPAS was mediated by gRNA. To achieve a high efficiency on PAS interference, gRNA should be designed to target the core regulatory elements of polyadenylation, such as PAS signal and upstream UGUA element, or the splicing sites in the case of splicing coupled APA such as intronic or alternative terminal exon APA. Whereas PAS signal defined by the canonical AAUAAA hexamer and its close variants could be identified for most PASs, there are about 10% of PASs without known PAS signal variants upstream of their cleavage sites. We demonstrated even for these PASs, gRNA designed to target the 30–60 nt upstream of the cleavage site, where known PAS signals were located, could efficiently inhibit the PAS usage. Beyond core elements, it was conceivable that CRISPR-iPAS could also be used for screening for other accessory elements by designing multiple tiling gRNAs targeted at region more distal to the targeted PAS. In addition to target site, the binding affinity between the gRNA and its target, which could affect the interfering efficiency on the targeting PAS, was another important factor to be considered for gRNA design. As shown for the reporter gene, the weak binding of gPAS due to low GC content of its target region resulted in a much lower repressive effect than that of gUSE with strong target binding affinity (Figure 1B–D).

Whereas CRISPR-iPAS could efficiently repress the usage of targeted PAS, it could also reduce the abundance of total mRNA from the same gene, although the latter effect was variable. On one hand, the perturbation of proximal PAS often led to the decrease of total mRNA abundance. In the case of tandem 3′UTR APA, the efficiency in repression of proximal PAS to a certain extent correlated with that in reduction of total mRNA abundance. On the other hand, no such effect could be observed for interference of distal PAS at all. The reason for the discrepant effects on total mRNA abundance observed for proximal versus distal PAS likely lied in the different fates of the mRNA after perturbation of its polyadenylation process. For proximal PAS, the interference of its polyadenylation process might result in RNAs with aberrant processing and/or elongation, which would be then degraded by RNA quality control system in the nucleus. In contrast, as elegantly demonstrated in a recent paper from Yu Zhou and Xiang-dong Fu's lab, the distal PAS was often processed first, and the resulting transcripts could serve as precursors for further processing at proximal PAS sites. Therefore, the perturbation of distal PAS might lead to the more efficient usage of proximal PAS at the cost of the distal one, but without negatively affecting the total mRNA abundance. Other factors might also contribute to the observed effect on total mRNA abundance. For example, the gRNA target region might contain, in addition to the polyadenylation regulatory elements, the binding sites of other RNA binding proteins (RBPs) which could affect RNA stability. Block the access by such regulatory RBPs would affect the RNA stability and thereby also the RNA abundance. Indeed, the effect exerted by the gRNA targeting the sequence outside of the core regulatory region in the proximal PAS of gene SMAD4 might be due to such mechanism. Moreover, given that the stability of different APA isoforms could vary, the shift in the proportion of APA isoforms by modulating PAS usage would lead to the apparent change in total RNA abundance.

With *IGF2BP1* as an example, we demonstrated that CRISPR-iPAS could be successfully applied to investigate the function of APA isoforms. However, in such functional study, experiments need to be carefully designed and data need to be interpreted with caution as well. For example, as discussed above, PAS perturbation might reduce the mRNA abundance. Therefore, the observed phenotypic effects might arise from the decrease of total mRNA level, instead of the differential function of the different APA isoforms. To ascertain the latter, experiments such as those measuring the RNA stability and translation efficiency, both for different isoforms and in cells with or without PAS perturbation, would be essential, as exemplified in our study of *IGF2BP1*. Finally, in addition to the detailed mechanistic study of individual APA isoforms, CRISPR-iPAS, in combination with appropriate cellular assays, could also enable systematic functional screening of thousands of different APA isoforms in mammalian cells, which would expand our knowledge of functional relevance of APA regulation to an unprecedented level.

## DATA AVAILABILITY

The sequencing data in this study have been deposited into the GEO database under accession number GSE162305.

## Supplementary Material

gkac108_Supplemental_Files
