# Supplementary material for: CRISPR-iPAS: a novel dCAS13-based method for alternative polyadenylation interference
Source: Nucleic Acids Res. 2022 Feb 22;50(5):e26. doi: 10.1093/nar/gkac108 (PMC8934656; doi:10.1093/nar/gkac108)
Supplement: gkac108_Supplemental_Files [file gkac108_supplemental_files.zip › Supplementary Table Legends.docx]

**Supplementary Table Legends**

## Supplementary Table 1. The Oligo sequences used in this study.

Sheet (1) Sequences of gRNA targets.

Sheet (2) Sequences of qPCR primers.

Sheet (3) 3’RACE primers.
