# Supplementary material for: CRISPR-iPAS: a novel dCAS13-based method for alternative polyadenylation interference
Source: Nucleic Acids Res. 2022 Feb 22;50(5):e26. doi: 10.1093/nar/gkac108 (PMC8934656; doi:10.1093/nar/gkac108)
Supplement: gkac108_Supplemental_Files [file gkac108_supplemental_files.zip › SupplementaryFigures.pdf]

Supplementary Figure 1

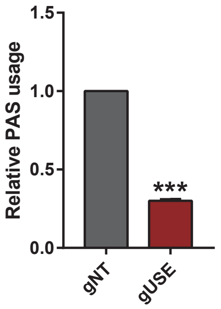

Supplementary Figure 1. The usage of the reporter PAS was measured by 3' end mRNA sequencing in HEK293T^{APA} reporter cells co-transfected with dPguCas13b and gUSE or gNT. Comparing with gNT, dPguCas13b with gUSE significantly inhibited the usage of the reporter PAS.

Two replicates were performed for the 3' end mRNAs sequencing. Error bars represent SEM., ***: $p < 0.001$, paired two-way Student's t-test.

Supplementary Figure 2

A

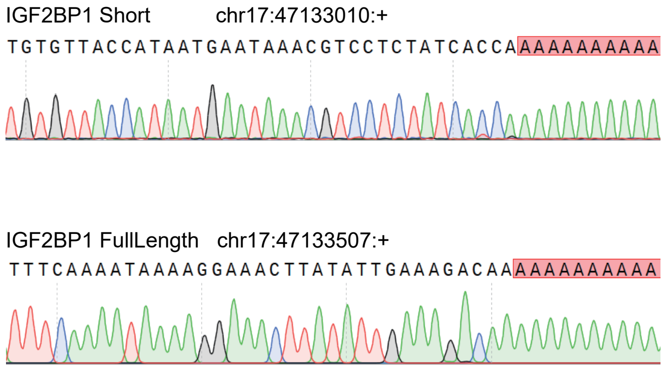

B

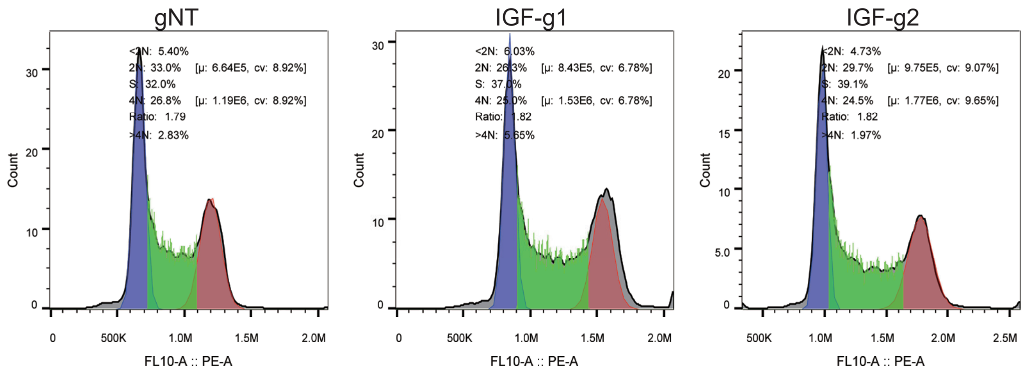

Supplementary Figure 2. (A) Sanger sequencing confirmed that the cDNA products derived from 3'RACE assay were APA isoforms ended with the proximal PAS (Short) and the distal PAS (Full Length).

(B) Flow cytometry showed that the perturbation of distal PAS usage by CRISPR-iPAS increased and decreased percentage of cells in S and G0/G1 phase, respectively.
